# Construction and validation of the indicator system for factors influencing pre-match tactical decision-making of volleyball coaches

**DOI:** 10.3389/fpsyg.2026.1804440

**Published:** 2026-06-19

**Authors:** Zheng Sun, Fan Zhang

**Affiliations:** 1 College of Physical Education, Anhui Normal University, Wuhu, China; 2 Department of Kinesiology, College of Education and Behavioral Sciences, Arkansas State University, Jonesboro, AR, United States

**Keywords:** coach, Delphi method, indicator system, influencing factors, pre-match tactical decision-making

## Abstract

**Introduction:**

The scientific and rational nature of volleyball coaches’ pre-match tactical decision-making is crucial for enhancing athletes’ performance and improving team tactical effectiveness. This study aimed to construct and preliminarily validate an indicator system for factors influencing volleyball coaches’ pre-match tactical decision-making.

**Methods:**

Based on systems theory and sports training and competition theory, this study constructed the indicator system by combining the Delphi method and the Precedence Chart method. The validity and practical applicability of the system were examined through expert retrospective validation and a questionnaire survey.

**Results:**

The expert retrospective coverage rate was 80.6%, indicating that the system comprehensively reflected the key factors influencing pre-match decision-making. The refined indicator system included four primary indicators: Tactical Decision Execution System (0.4375), Tactical Decision-Making Subject System (0.3125), Match Evaluation System (0.0625), and Organizational and Information Support System (0.1875). It also included nine secondary indicators and 51 tertiary indicators. Spearman correlation coefficients for all levels of indicators indicated strong consistency, demonstrating a high degree of alignment between expert and grassroots coaches’ decision-making perceptions.

**Discussion:**

This study provides theoretical support and a data-based framework for volleyball coaches’ pre-match information collection, data analysis, and match planning. It also offers a reference framework for coaches’ decision-making in other team sports.

## 1 Introduction

Volleyball is an intensely competitive and complex sport, characterized by its confrontational nature and strategic gameplay (Marcelino et al., 2011). The outcome of a match depends on a variety of subjective and objective factors, as well as the effective mobilization of forces. The key challenge lies in how to convert potential forces into tangible forces directly impacting the match, a process that requires coaches to make scientific and rational tactical decisions before the game. Pre-match tactical decision-making refers to the process in which the coach systematically arranges tactics, lineup combinations, substitution plans, and related details prior to the match. As a critical component of the coaching decision-making system, this process involves the interaction of multiple factors, including the coach, match objectives, opponent information, match rules, and the environment. It plays a crucial role in ensuring the smooth progression of the game. Its scientific and rational nature directly influences the performance of individual athletes and the overall strength of the team, thereby affecting the achievement of match objectives.

Every decision is influenced by multiple factors, and coaches’ pre-match planning is no exception. It involves not only tactical arrangements but also player allocation, psychological preparation, opponent analysis, and several other dimensions. A literature review reveals that existing research primarily focuses on the following five aspects.

### 1.1 Shift in research perspective: viewing “pre-match planning” as a measurable decision-making process

For a long time, coaching tactical decision-making has been placed within the framework of “contextual command or on-the-spot response,” with the common premise being that decisions are not based on linear reasoning, but rather involve weighing risks, benefits, and feasibility under time pressure and incomplete information conditions (Jones and Wallace, 2005; Kaya, 2014; Harvey et al., 2015). However, in high-level volleyball, there is also a crucial “decision chain” during the pre-match phase: opponent scouting and information integration (Gesbert et al., 2016; Palao and Hernández-Hernández, 2014), lineup and role allocation (Fiander et al., 2023), training load and recovery regulation (Debien et al., 2018), contingency planning and risk management (Gabbett, 2016). These decisions preemptively limit the space for adjustments and the potential benefits during the match. Therefore, defining the pre-match phase as an independent research object helps to translate the key constraints of the decision-making process (such as situational complexity, time pressure, and prior conditions) into measurable tasks and indicators (Ashford et al., 2021), providing a starting point for subsequent modeling.

### 1.2 Decision-making subject: expert recognition ability, efficacy beliefs, and emotional regulation

Regarding how high-level coaches form high-quality pre-match plans, research has shifted from focusing on “knowledge stock” to the expert cognitive mechanism of “recognition—matching—generation.” Drawing on the findings of Harvey et al. (2015) and Gründel et al. (2013), in dynamic and time-constrained match contexts, expert coaches tend to rely on experience to recognize situational patterns, prioritize key attractors, and generate an initial intervention plan. They then make necessary evaluations and adjustments, rather than conducting an exhaustive, option-by-option comparison. This means that the core of pre-match information processing lies not in the “amount of information” but in the “clue selection rules.” Meanwhile, the complexity of the competitive environment can amplify cognitive load and emotional fluctuation risks. Without a stable psychological regulation mechanism, pre-match plans are more likely to result in seemingly complete but practically unmanageable non-optimal choices (Kaya, 2014). On this basis, coaching efficacy can be seen as an important psychological structure connecting the coach’s experience and supportive resources, efficacy beliefs, and coaching behaviors. The sources of efficacy information are multidimensional and correlated with the coach’s teaching and training organization behaviors (Feltz et al., 1999; Myers et al., 2005; Myers et al., 2017). Additionally, emotional intelligence affects the quality and effectiveness of coaching interventions, as it can reduce emotional interference under pressure and improve decision-making stability and rationality (Teques et al., 2019; Zajonz et al., 2024).

### 1.3 Decision-making constraints: training load—recovery—injury risk and player availability

High-level volleyball pre-match tactical decisions are often initially constrained by “player availability”: who can play, how long they can play, and the level of risk. Debien et al. (2018) demonstrated through longitudinal monitoring that volleyball players’ internal training load and recovery levels fluctuate significantly and affect key physical performance, such as jumping, indirectly influencing lineup selection and tactical risk preferences. At the same time, Fox et al. (2018) found that the relationship between load and performance is not always linear and stable, suggesting that without a reasonable framework for interpreting indicators, pre-match tactical decisions may lead to “pseudo-precise” judgments. From a risk management perspective, a rapid increase in short-term load and poor scheduling are major sources of injury risk, and pre-match tactical deployment needs to incorporate a “risk–benefit” decision-making framework (Gabbett, 2016). Specifically, in volleyball, injury epidemiology studies show that there is a considerable proportion of acute and overuse injuries during the season (Aagaard et al., 1997; Verhagen et al., 2004). In high-intensity matches, mental and muscle fatigue can lead to slow reactions and imprecise movements, thus affecting tactical execution and overall performance (Coutinho et al., 2018). An athlete’s injury status also influences tactical adjustments. If key players are injured, the coach must adjust the lineup or tactical strategy to accommodate personnel changes or the player’s physical condition (Surya et al., 2015). Furthermore, external factors such as the match environment (Aughey et al., 2013), referee decisions (Avugos, 2024), and audience behavior (Bilalić et al., 2021; Oldfield et al., 2026) also affect the coach’s pre-match tactical decisions, requiring the coach to prepare appropriate countermeasures in advance.

### 1.4 Evidence tools: opponent scouting, match analysis, and data-driven pre-match preparation

With the integration of match analysis and data science methods into the preparation process, pre-match preparation is evolving from experience-based judgment to an “explainable evidence chain.” On the one hand, research on competition performance has demonstrated a stable relationship between match outcomes and key technical aspects: for example, in high-level competitions, tactical performance varies with the opponent’s strength and the match situation, necessitating that pre-match plans include both “opponent strength” and “situational planning” (Marcelino et al., 2011, 2012). On the other hand, research on set/match unit indicators provides directly transferable candidate variables for the “indicator system,” such as match-deciding indicators in typical sets, the discriminating role of key techniques on match outcomes, and the critical link of “serve-receive—setter organization—offensive conversion” (Drikos and Vagenas, 2011; Peña and Casals, 2016; González-Silva et al., 2020). Furthermore, Medeiros et al. (2014) found that situational factors such as technical selection, technique type, age, and role collectively influence serve and attack efficiency, suggesting that pre-match deployment should incorporate situational variables and adjust the focus and priority of “targeted arrangements” accordingly. Based on this, de Leeuw et al. (2022) further advanced data analysis to the modeling level, using machine learning to link jumping load and strength training characteristics to match performance, providing more operational evidence for training plan adjustments and pre-match preparation.

### 1.5 Decision implementation: leadership style, coach-athlete relationship, and “feasibility of execution”

The value of pre-match planning lies not only in “what has been designed,” but also in “whether it can be executed effectively by the team.” From a team perspective, there is a stable association between transformational leadership, team cohesion, and performance level, with cohesion serving as the social-psychological foundation for the implementation of tactical plans (Callow et al., 2009). In youth team settings, leadership style and the quality of the coach-athlete relationship jointly shape team success and positive developmental experiences. This suggests that the communication approach, role delegation, and relational atmosphere of pre-match plans will influence execution willingness and collaboration quality (Vella et al., 2013). From a motivational mechanism perspective, the coach-athlete relationship can affect athlete motivation and engagement through pathways such as autonomy support and feedback methods, thereby altering the acceptance and execution intensity of tactical arrangements (Mageau and Vallerand, 2003). Furthermore, Lafrenière et al. (2011) found a link between passion/engagement experiences and interaction quality, with positive psychological factors being highly correlated with sports participation experiences, meaning that the “psychological feasibility” of pre-match tactical plans (e.g., trust, engagement, and identification) should be included in the indicator system, rather than treated merely as background variables.

In existing research, Sun and Zhang (2025) used the Delphi method to summarize and extract the influencing factors of volleyball coaches’ in-game tactical decisions, providing a clear entry point for future studies. However, in-game and pre-match tactical decisions should not be treated as the same decision task at different time points. In-game decisions are mainly based on dynamic information that emerges during match play, such as score changes, opponent adjustments, tactical execution, and real-time athlete performance. By contrast, pre-match decisions rely more on relatively stable and pre-obtainable information, including opponent scouting, player availability, lineup planning, training-load status, recovery, and risk preparation. Therefore, the pre-match phase represents a distinct decision subsystem with its own information structure, cognitive demands, and practical functions. Despite this distinction, discussions on the factors influencing volleyball coaches’pre-match decisions remain relatively fragmented. Relevant studies are mostly found in empirical summaries or case descriptions, with limited attention to structured theoretical frameworks and actionable measurement dimensions. Existing research also tends to treat coaching decision-making as a unitary process, giving insufficient attention to how multiple factors interact before the match to shape tactical planning. This limitation weakens the explanatory value of existing theory and reduces its usefulness for coaching practice.

To address this research gap, this study aimed to construct an indicator system for factors influencing volleyball coaches’ pre-match tactical decision-making and to preliminarily examine its practical applicability. In this study, pre-match tactical decision-making was understood as a hierarchical decision system shaped by system elements, competition context, and training status, rather than as a simple collection of influencing factors. System elements mainly involve decision actors, execution resources, and information support; competition context refers to match evaluation and external conditions; and training status concerns athletes’ competitive ability, physical condition, recovery, and the basis for tactical execution. The Delphi method, Precedence Chart method, expert backtracking, and questionnaire survey were used to systematically screen relevant factors, determine indicator weights, and assess practical applicability. This process produced an indicator framework covering athletes, coaches, match evaluation, and organizational and information support. The proposed system may provide a structured basis for coaches’ pre-match information collection, tactical analysis, and match planning. For novice and reserve coaches, it may also serve as a practical reference tool for identifying the key elements and priorities of pre-match decisions, thereby reducing reliance on trial-and-error experience alone.

## 2 Research methods

### 2.1 Delphi method

The Delphi method is a systematic approach for obtaining expert consensus by inviting experts from relevant fields to participate in an anonymous multi-round survey to collect their opinions (Hsu and Sandford, 2007). After each round, the experts’ feedback is summarized and anonymously returned to the participants, allowing them to adjust or confirm their views based on the group’s feedback until consensus is reached or the majority opinion stabilizes. Due to its anonymity, multi-round feedback, and statistical integration mechanisms, the Delphi method is widely applied in health sciences and sports research, particularly in the development of evaluation systems, decision-making criteria, and consensus frameworks (Okoli and Pawlowski, 2004). Common applications include physical fitness assessments, behavioral evaluations, and sports intervention standards (Ye et al., 2024; Chen et al., 2024).

In this study, we employed the Delphi method to construct an indicator system for volleyball coaches’ tactical decision-making. This method effectively integrates experts’ practical experience and judgment, making it particularly suitable for research scenarios where empirical data is lacking and the decision-making factors are complex and dependent on expert experience.

#### 2.1.1 Expert panel composition

This study follows the principles of combining representativeness and authority, with voluntary participation from experts. Based on the research needs, the selection criteria were established: ① possessing substantial theoretical research achievements or over 10 years of relevant practical experience; ② holding a senior professional title or being a current provincial-level volleyball team head coach with a senior coaching qualification or above; ③ having an in-depth understanding of volleyball competition and coaches’ tactical decision-making; ④ willing to participate in this research and demonstrating a high level of enthusiasm. There is no unified standard for determining the number of experts in a Delphi study in the existing literature. Some studies suggest that in Delphi research involving interdisciplinary, multi-field experts or multiple stakeholders, the optimal number of experts for the group should be between 60 and 80. This scale helps improve the reproducibility of the research results (Manyara et al., 2024). In Delphi studies focused on a single field, the expert group typically consists of 8 to 20 experts (Paul, 2008; Jorm, 2015). Furthermore, some studies argue that the size of the Delphi expert group should be determined based on time and economic conditions, with an ideal number of 8 to 23 experts, which is considered practical and feasible (Shang, 2023).

Based on the selection criteria, a convenience sampling method was used to contact and invite 15 consulting experts. Ultimately, 12 experts completed the consultation survey (Table 1). The expert panel was designed to include both research-oriented and practice-oriented expertise. Six experts were university professors with long-term research experience in volleyball competition theory, technical-tactical analysis, teaching and training, and performance analysis, and they mainly provided academic judgment from theoretical and methodological perspectives. The other six were current head coaches of provincial-level or higher volleyball teams, all holding senior coaching qualifications and having extensive experience in team management, pre-match preparation, match coaching, and tactical decision-making. Among the six practice-oriented coaching experts, four were head coaches of men’s volleyball teams and two were head coaches of women’s volleyball teams, providing practical judgment based on high-level coaching experience. This composition helped the indicator system draw on both theoretical interpretation and coaching experience, thereby improving its content coverage and practical relevance.

**Table 1:** Basic information on consulting experts (*N* = 12).

Number	Gender	Title	Research or Area of Expertise
1	Male	Professor	Volleyball performance analysis
2	Male	Professor	Basic theories of volleyball and teaching and training
3	Male	Professor	Volleyball theory and practice
4	Male	Professor	Volleyball teaching and training
5	Male	Professor	Volleyball teaching and training
6	Male	Professor	Volleyball teaching and training
7	Male	Senior coach	Head coach
8	Male	National-level coach	Head coach
9	Male	National-level coach	Head coach
10	Male	Senior coach	Head coach
11	Male	National-level coach	Head coach
12	Male	National-level coach	Head coach

#### 2.1.2 Consultation methods and procedures

Based on an extensive review of relevant domestic and international literature, in-depth interviews, and field research, a consultation questionnaire was developed for constructing the indicator system for volleyball coaches’ pre-match tactical decision-making factors. The questionnaire adopted a Likert 5-point scale, with scores ranging from 1 to 5 to represent the importance of each influencing factor. Two rounds of expert consultation were conducted.

Round 1 Consultation: The questionnaire was sent to consulting experts via email and WeChat, aiming to collect their feedback on the structure and content of the indicator system, as well as to obtain the experts’ ratings for the indicators. This round provided foundational data to further refine the indicator system.

Round 2 Consultation: After collecting the responses from the first round of the questionnaire, expert feedback was organized, and the indicator system was revised based on their comments. The maximum, minimum, and mean values of each indicator were calculated, along with the percentage distribution of each score. These data, along with the ratings provided by each expert and the consolidated opinions of the entire expert group, were incorporated into the second-round questionnaire for further consultation.

#### 2.1.3 Expert engagement, authority, and consensus

The experts’ engagement level is typically measured by the questionnaire response rate. A total of 15 questionnaires were distributed in the first round, with an effective response rate of 80%. In the second round, 12 questionnaires were distributed, achieving a 100% response rate, indicating that the consulting experts demonstrated a high level of participation in the study.

The authority level of the experts is represented by the authority coefficient (Cr), which ranges from 0 to 0.95. A higher value indicates greater authority (Eklund et al., 2007). Based on the statistical data regarding the experts’ judgment basis and familiarity, the authority coefficient was calculated as Cr = 0.895, indicating that the consulting experts possess a high level of authority and a deep understanding of the research topic, making their opinions highly credible.

The degree of consensus among the experts’ opinions is measured by the coefficient of variation (Cv) and Kendall’s concordance coefficient (W). Typically, an average score greater than 3.50 and a Cv value less than 0.25 are considered within an acceptable range (Wang et al., 2021; Xie et al., 2023). Kendall’s W ranges from 0 to 1, measuring the degree of concordance among multiple ranked variables. The statistical results show that the Cv for first-level indicators ranged from 0 to 0.14, for second-level indicators from 0 to 0.15, and for third-level indicators in the first round from 0 to 0.27 and in the second round from 0 to 0.20, with an average value of 0.12. The concordance coefficients W for each level of indicators were 0.722, 0.671, and 0.400, respectively. Chi-square tests showed that the *p*-values for all coefficients were less than 0.01 (Table 2), indicating a high level of consistency in the experts’ opinions.

**Table 2:** Coordination coefficient statistics for each level of indicators in the second round of expert opinions.

Statistic	Primary indicators	Secondary indicators	Tertiary indicators
Number of indicators	6	15	72
Coordination coefficient (W)	0.722	0.671	0.400
Chi-square value (χ^2^)	43.313	112.764	355.340
Asymptotic significance (p)	0.000	0.000	0.000

### 2.2 Precedence chart method

The Precedence Chart (PC) is a method for determining indicator weights through pairwise comparisons (Wang and Tian, 2016). It was selected in this study because it can derive relative priorities from Delphi consensus scores without requiring experts to complete another large set of pairwise judgments. Based on the experts’ ratings, the mean score of each indicator is calculated. The mean scores are then compared in pairs. A value of “1″ indicates that an indicator is more important, “0″ indicates less importance, and “0.5″ indicates equal importance. These comparisons form a weight calculation matrix. The relative score of each indicator (TTL) is obtained from the matrix. The weight of each indicator is computed as its TTL divided by the sum of TTL values across all indicators. The PC method also includes a complementary check to examine the internal consistency of the pairwise comparison results. Because the weighting procedure was conducted after two Delphi rounds, the retained indicators had reached acceptable levels of mean scores, coefficients of variation, and Kendall’s W agreement, providing a relatively stable expert-consensus basis for the weighting process.

### 2.3 Survey method

Based on expert-refined practical-level indicator system, a validation questionnaire was developed, containing 64 items (4 first-level indicators, 9 second-level indicators, and 51 third-level indicators) using a 5-point Likert scale. This questionnaire was designed to examine whether the priority rankings derived from the expert-refined practical-level indicator system were consistent with those of grassroots coaches. Twelve experts who participated in the Delphi method were invited to assess the validity of the questionnaire. Among them, 10 experts considered the structure and content of the questionnaire highly reasonable, while 2 experts deemed it reasonable. It should be noted that the questionnaire was used to examine consistency in practical priority rankings, rather than to provide final structural or predictive validity evidence.

The questionnaire was distributed using snowball sampling. It was shared mainly through coach WeChat groups affiliated with the Volleyball Branch of the Chinese Students’ Sports Federation (universities), or distributed individually. A total of 67 valid responses were collected. Among the 67 participating coaches, 46 were head coaches of men’s volleyball teams and 21 were head coaches of women’s volleyball teams, accounting for 68.7 and 31.3% of the valid sample, respectively. Statistical results showed that the Cronbach’s *α* coefficients for the first, second, and third-level indicators were 0.812, 0.847, and 0.923, respectively, indicating good reliability and providing a solid foundation for subsequent research.

## 3 Results and analysis

### 3.1 Construction of the indicator system

#### 3.1.1 Theoretical basis for indicator selection

The construction of the indicator system primarily follows four key principles: scientificness, systematization, comprehensiveness, and hierarchy. It is guided by the core question, “How do volleyball coaches make pre-match tactical decisions?” The factors closely related to coaches’ pre-match tactical decision-making are selected, and the theoretical basis for these factors includes systems theory, sports competition theory, and sports training theory.

Systems Theory: From a systems theory perspective, volleyball competition is a complex dynamic system. There are diverse and extensive interrelations and interactions between the various elements within the system, and these elements are inevitably related to tactical decision-making. The characteristics and changes of certain elements in the system directly influence the coach’s pre-match tactical decisions.

Sports Competition Theory: Sports competition is a relatively broad concept, encompassing the management activities of event organizers, the officiating activities of referees, the participation activities of athletes, coaches, and their teams, and even the involvement of spectators and media. These elements undoubtedly have a significant impact on the coach’s pre-match tactical decisions during a volleyball competition.

Sports Training Theory: The changes in and performance patterns of athletes’ competitive abilities, the determining factors of athletic performance, and the elements of competitive potential all influence the coach’s pre-match tactical decisions.

#### 3.1.2 Establishment of the indicator pool

Using methods such as analysis and synthesis, factors influencing coaches’ pre-match tactical decision-making were selected from sources such as books, textbooks, journal articles, and other relevant literature. Some “meta-indicators” were further broken down and revised. The primary sources of literature were books and journal articles in the fields of sports training, sports competition, competition preparation, and match strategies. The extraction of indicators was mainly carried out through the following approaches: drawing on existing indicators from previous studies, adapting indicators from other sports, adopting indicators proposed by experts, suggesting new indicators based on personal cognition, and extracting indicators from in-depth interview materials. Based on this, the indicator pool was constructed in stages and steps. During repeated reviews and checks, all indicators were comprehensively processed: duplicate or highly similar indicators were removed, weakly correlated indicators were excluded, and some indicators were renamed. In the end, 129 relevant influencing factors were identified, forming the final indicator pool.

#### 3.1.3 Construction of the hierarchical system

**Figure 1:**
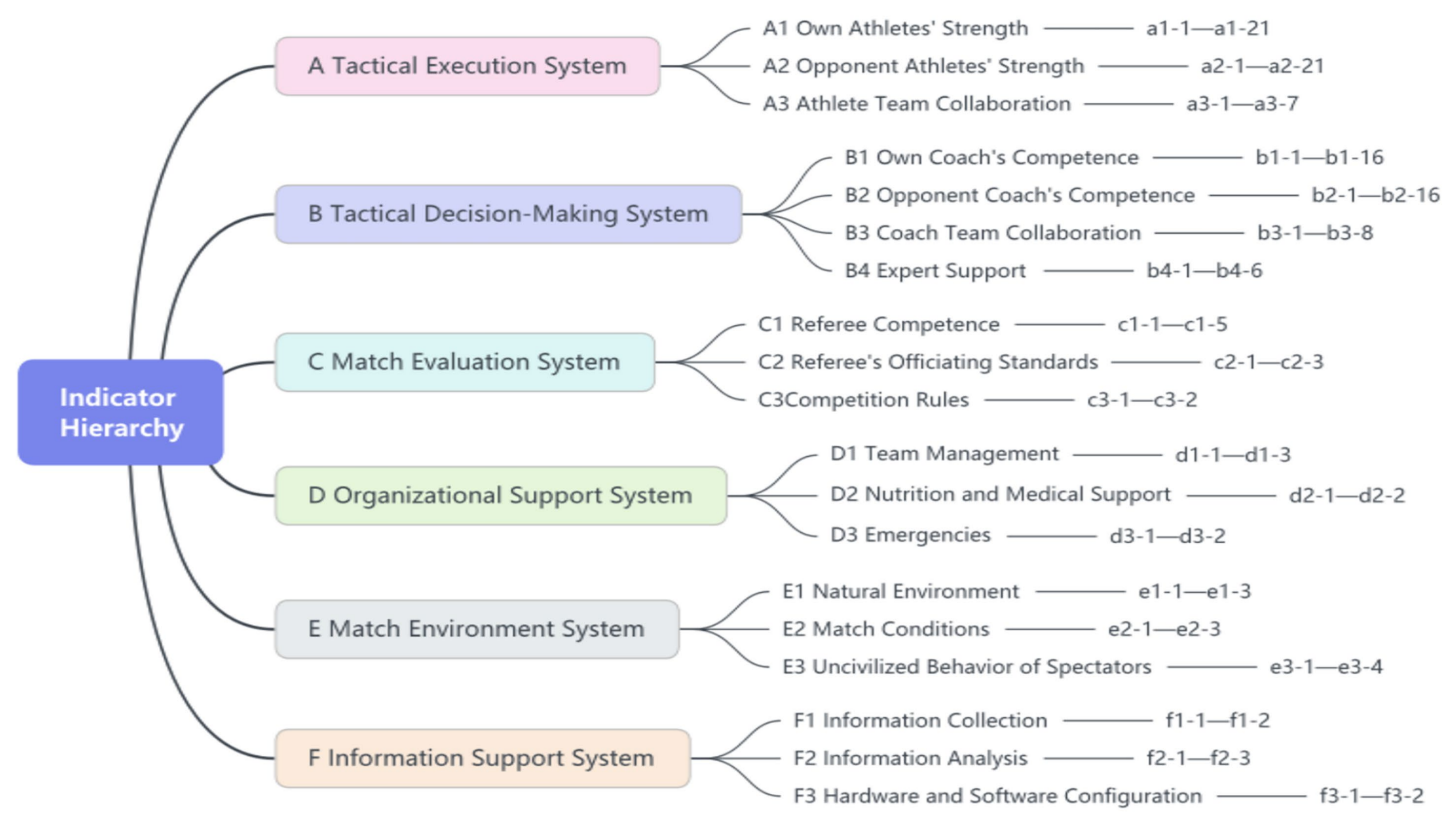
Hierarchical structure of the volleyball coach’s pre-match tactical decision-making indicator system.

To enhance the structural clarity of the indicator system, this study organized the indicators hierarchically according to the logic that pre-match tactical decision-making moves from broad systems to specific factors. Primary indicators mainly represent the core system dimensions involved in pre-match tactical decision-making. Secondary indicators describe the functional modules within each system dimension, whereas tertiary indicators refer to influencing factors that coaches can identify, assess, and use during pre-match preparation and tactical planning. According to the principles and basic requirements for determining indicators, the indicators in the pool were categorized and classified using a comprehensive approach to clarify the hierarchical and inclusion relationships among indicators and to avoid redundancy or content confusion. During this process, indicators with overlapping meanings, similar wording, or unclear boundaries were merged, renamed, or removed after expert discussion, so that each retained indicator could represent a relatively distinct aspect of pre-match tactical decision-making. In the specific process, indicators related to a particular aspect or category were classified, with A, B, C, etc., representing the first-level indicators. Corresponding second-level indicators were denoted by the first-level indicator letter followed by a number (A1, A2, etc.), and third-level indicators were represented by lowercase letters and numbers (a1-1, a1-2, etc.). At the same time, the first-level and second-level indicators were named, and the preliminary hierarchical structure of the indicator system was constructed. Ultimately, an indicator system was formed, comprising 6 first-level indicators, 19 second-level indicators, and 129 third-level indicators (Figure 1).

#### 3.1.4 Indicator selection

1 Expert-based selection

To ensure the scientific and rational nature of the indicator system, five experts and coaches in the field of volleyball were invited to evaluate the structural validity, content rationality, relevance, and wording expression of the indicator system, as well as to conduct an expert-based selection of the indicators. The results indicated that the first-level, second-level, and third-level indicators for volleyball coaches’ pre-match tactical decision-making factors were reasonable in terms of classification, naming, and content structure. However, further standardization of the wording expression was needed, and some duplicate indicators or those with low impact needed to be removed.

Regarding the structure and content of the indicator system, experts suggested the following changes: the first-level indicators “A Tactical Execution System” and “B Tactical Decision-Making System” should be renamed as “A Tactical Decision-Making Execution System” and “B Tactical Decision-Making Subject System,” respectively. Additionally, the second-level indicators “B2 Opponent Coach Competence” and “C1 Referee Competence” and their corresponding third-level indicators were deleted. The rationale for this decision is that the “Opponent Coach Competence” has low relevance to the coach’s pre-match tactical decision-making and is difficult to assess effectively. “Referee Competence,” particularly in modern volleyball matches where technology and information systems are increasingly prevalent, has limited impact and weak correlation with the coach’s pre-match decisions, making it similarly difficult to quantify.

Several third-level indicators were merged, renamed, or deleted (Table 3). After adjusting according to the expert-based selection feedback, the indicator system now includes 6 first-level indicators, 17 second-level indicators, and 92 third-level indicators.

2 Expert panel discussion selection

**Table 3:** Indicators processed based on expert opinions from indicator screening.

**Indicator**	**Suggestions or opinions**	**Resolution**
A, B, a1-5, a1-16, a2-5, a2-16, b1-6	Modify indicator names	Modify
B2, C1	Delete	Delete
a1-1 and a1-3, a1-2 and a1-4, a2-1 and a2-3, a2-2 and a2-4, b1-11 and b1-12	“Personal Technical Skills” and “Personal Tactics” are merged into “Personal Technical-Tactical Skills”, "Specialized Techniques” and “Tactics” are merged into “Specialized Technical-Tactics”	Merge
a1-9, a2-9, b1-4, b2-1—b2-16, b3-2, b3-5, b3-6, b3-7, b3-8, b4-1, b4-3, c1-1—c1-5, e3-4	Indicators that have weak similarity or low correlation with other indicators are removed	Delete

Building on the expert-based selection, three experts were invited to participate in a group discussion to further refine the indicator system. The revisions suggested by the expert panel discussion are as follows (Table 4): No changes were made to the first-level indicators.

**Table 4:** Indicators processed based on the group experts’ discussion.

**Indicator**	**Suggestions or opinions**	**Resolution**
C3	Modify the indicator names to “Competition Organization”	Modify
a3-1, a3-2, a3-3, a3-4, b1-1	Add “Own” before the original indicator names; change “Knowledge Reserve” to “Professional Knowledge Reserve”	Modify
a1-15 and a1-16, a2-15 and a2-16, b1-6 and b1-8, b1-9 and b1-10, b1-13 and b1-14, e1-1 and e1-2, e2-1 and e2-2, f1-1 and f1-2, f2-2 and f2-3	Merge “Understanding of Referee’s Officiating and Competition Rules,” “Coach’s Understanding and Trust in Athletes,” “Coach’s Information Selection and Analysis,” “Climate and Weather,” “Venue and Equipment,” “Information Collection Methods and Approaches,” and “Information Screening and Integration.”	Merge
a1-11, a1-14, a2-11, a2-14, b3-3, b4-6, c2-3	Indicators that have weak similarity or low correlation with other indicators are removed	Delete

For the second-level indicators, it was recommended to merge “F1 Information Collection” and “F2 Information Analysis,” as they represent a continuous process. Additionally, the indicators “E1 Natural Environment” and “E23 Competition Conditions” were considered to have minimal impact on coaches’ pre-match tactical decision-making in current training and competition conditions, and thus it was suggested to merge them to reduce the number of second-level indicators.

For the third-level indicators, some content needed to be merged, streamlined, or clarified with additional wording to ensure the accuracy of the indicator names and avoid ambiguity. Some indicators were also renamed to improve their precision.

After the adjustments and reordering, the preliminary indicator system for volleyball coaches’ pre-match tactical decision-making factors was finalized, comprising 6 first-level indicators, 15 second-level indicators, and 76 third-level indicators.

#### 3.1.5 Revision and improvement of the Indicator system

Based on the expert-based selection and panel discussion, a formal survey questionnaire for the volleyball coach pre-match tactical decision-making factor indicator system was developed. The system was further evaluated using the Delphi method and the threshold method, ultimately determining the final indicator system.

The first-level and second-level indicators were primarily revised based on the arithmetic mean (Mj) and the coefficient of variation (Cv). Indicators with Mj ≥ 3.50 and Cv ≤ 0.25 were retained, while those that did not meet these standards were deleted. The results from the two rounds of Delphi expert surveys showed unanimous expert ratings, with no changes made to the original indicators. Statistical results indicated that the mean values (Mj) for the first-level indicators ranged from 3.75 to 5, with an average of 4.15, and the Cv values ranged from 0 to 0.14. For the second-level indicators, the mean values (Mj) ranged from 3.50 to 5, with an average of 4.19, and the Cv values ranged from 0 to 0.15. Consistency tests showed that the coordination coefficients (W) for the first-level and second-level indicators were 0.722 and 0.671, respectively, with *p*-values of 0.00 (Table 2), indicating a high degree of agreement among the consulting experts and confirming the selection of all indicators.

The number of third-level indicators was relatively large, so the threshold method was used to refine the third-level indicators. The threshold method calculates the threshold based on each indicator’s maximum score frequency, mean value, and coefficient of variation (Table 5), and deletes indicators with relatively low importance. The calculation method for the threshold is as follows: for the maximum score frequency and mean value, the threshold is calculated as “Threshold = Mean - Standard Deviation,” and for the coefficient of variation, the threshold is calculated as “Threshold = Mean + Standard Deviation.” To avoid mistakenly removing important indicators, only those indicators that meet the condition of “maximum score frequency and mean greater than the threshold, and coefficient of variation lower than the threshold” were retained, while indicators not meeting these criteria were deleted.

**Table 5:** Threshold table from two rounds of expert consultation.

**Round**	**Statistic**	**Mean**	**Standard deviation**	**Threshold**
Results of the first round of expert consultation	FSF	0.4748	0.2536	0.2212
	M	4.35	0.37	3.98
	CV	0.1371	0.0509	0.1880
Results of the second round of expert consultation	FSF	0.4755	0.2834	0.1921
	M	4.38	0.39	3.99
	CV	0.1224	0.0438	0.1662

Following the indicator selection criteria, during the first round of screening, indicators with a maximum score frequency lower than 0.22, a mean value lower than 3.98, and a coefficient of variation (Cv) greater than 0.19 were excluded. The results showed that only “e1-1 Weather Characteristics” met the exclusion criteria (Kj = 0.17, Mj = 3.50, Cv = 0.26).

In the second round of screening, indicators with a maximum score frequency lower than 0.19, a mean value lower than 3.99, and a Cv greater than 0.17 were excluded. A total of 3 indicators were removed, namely:

“a2-5 Opponent Athletes’ Professional Knowledge Reserve” (Kj = 0.17, Mj = 3.75, Cv = 0.20).

“e2-3 Competition Venue and Equipment Conditions” (Kj = 0.08, Mj = 3.50, Cv = 0.19).

“e3-2 Audience Verbal Aggression” (Kj = 0.00, Mj = 3.50, Cv = 0.19).

After these revisions, the final indicator system for volleyball coaches’ pre-match tactical decision-making factors was determined, consisting of 6 first-level indicators, 15 second-level indicators, and 72 third-level indicators (Supplementary material 1).

### 3.2 Determination of indicator weights

Following the basic paradigm of the Precedence Chart (PC) method for determining indicator weights, the average values for each indicator were calculated based on expert ratings. These average values were then compared pairwise to construct the Precedence Chart weight calculation matrix (Table 6). Subsequently, the scores for each indicator were summed horizontally, and the relative scores (TTL values) for each indicator were calculated. These TTL values were then normalized to determine the weight of each indicator (Supplementary material 1).

**Table 6:** Weight calculation table for primary indicators based on the Precedence Chart method.

**Mean**	**Item**	**A Tactical execution system**	**B Tactical decision-making system**	**C Match evaluation system**	**D Organizational support system**	**E Match environment system**	**F Information support system**
5	A tactical execution system	0.5	1	1	1	1	1
4.667	B tactical decision-making System	0	0.5	1	1	1	1
3.75	C match evaluation system	0	0	0.5	0	1	0
4.083	D organizational support system	0	0	1	0.5	1	1
3.583	E match environment system	0	0	0	0	0.5	0
3.833	F information support system	0	0	1	0	1	0.5

### 3.3 Practice-oriented preliminary validation of the indicator system

#### 3.3.1 Expert backtracking validation

The backtracking method emphasizes reverse verification of influencing factors starting from practical contexts to improve the consistency between theoretical constructs and real-world decision-making. To test the applicability and content coverage of the constructed indicator system for volleyball coaches’ pre-match tactical decision-making factors in real decision-making scenarios, this study invited five head coaches from the Delphi consultation experts to conduct a contextual backtracking assessment.

Given that third-level indicators are most directly related to the operational aspects of pre-match tactical decision-making, the evaluation focused on these third-level indicators. During the process, each third-level indicator was transformed into a contextualized item (e.g., “In your pre-match tactical decision-making, do you consider the factor a1-1 Athlete’s individual technical and tactical ability?”). Coaches were asked to assess and select based on their actual pre-match tactical decision-making experiences, and to add any potentially missing factors in the open-ended section.

In data processing, to improve the interpretability of the screening rules, this study used the Item-level Content Validity Index (I-CVI) to quantify the backtracking results, calculated as follows: I-CVI = Number of coaches who agreed with the item / 5 (Lynn, 1986; Polit et al., 2007).

Given that this step was intended to pre-screen the contextual applicability of the third-level indicators rather than to replace the expert consensus formed through the Delphi process, an I-CVI threshold of ≥ 0.80 was used as the criterion for practical relevance (Davis, 1992; Zamanzadeh et al., 2015). In the present study, this threshold corresponded to agreement by at least four of the five backtracking experts. This criterion was selected to retain indicators supported by a clear majority of experts while avoiding an overly strict requirement of full agreement, which might remove contextually meaningful indicators because of individual differences in coaching experience or decision-making style. Thus, the threshold balanced the need to avoid excessive simplification and information loss with the need to remove low-frequency or less actionable items. Given the small backtracking sample, this threshold should be interpreted as an exploratory pre-screening criterion rather than as final validity evidence.

The results showed that the coverage rate for core third-level indicators (percentage of items selected by at least one coach) was 80.6%. No new category dimensions or substantial new items were identified within the backtracking sample, suggesting that the indicator system has good content coverage and applicability to pre-match tactical decision-making contexts.

Based on these results and expert suggestions, 21 third-level indicators were deleted. Additionally, considering conceptual overlap and practical indistinguishability, the first- and second-level indicators were integrated and optimized. This process reduced the six first-level indicators to four and the 15 second-level indicators to nine (Table 7).

**Table 7:** Indicators at each level processed based on expert backtracking results.

**Indicator level**	**Processing results**
Primary indicator	Delete E, merge D and F
Secondary indicator	Delete B3, D2, D3, E1, E2, F2, and modify F2 to D2
Tertiary indicator	Delete a1-5, a1-10, a1-11, a2-4, a2-9, a2-13, b3-1, b3-2, b3-3, c1-2, d1-3, d2-1, d2-2, d3-1, d3-2, e1-2, e1-4, e2-1, e2-3, f2-1, f2-2, Modify f1-1, f1-2, f1-3 to d2-1, d2-2, d2-3

To ensure that the revised indicator system’s weight structure aligns with the new indicator set, the weights for the practical level were not re-assigned but instead, weight transmission and normalization were performed based on the theoretical level weights. The weights for the retained third-level indicators remained the same, while the weights for the merged second- and first-level indicators were derived by summing the weights of the retained third-level indicators within them. These weights were then normalized at each level so that the total weight for each level equals 1 (Table 8).

**Table 8:** Weight calculation table for primary indicators based on the Precedence Chart method.

**Primary indicator**	**Secondary indicator**	**Tertiary indicator**
A Tactical execution system (0.4375)	A1 Own athlete’s strength (0.2099)	a1-1 Athlete’s personal technical-tactical ability (0.0388)
		a1-2 Athlete’s personal technical-tactical characteristics (0.0369)
		a1-3 Athlete’s physical condition (0.0281)
		a1-4 Athlete’s personality and psychological traits (0.0073)
		a1-6 Athlete’s physical fitness (0.0311)
		a1-7 Athlete’s recent competitive state (0.0281)
		a1-8 Athlete’s self-regulation ability (0.0227)
		a1-9 Athlete’s team awareness (0.0381)
		a1-12 Athlete’s age structure (0.0008)
		a1-13 Athlete’s competition experience (0.0281)
		a1-14 Athlete’s injury status (0.0112)
		a1-15 Athlete’s tactical execution ability (0.0350)
	A2 Opponent athlete’s strength (0.1605)	a2-1 Opponent’s athlete’s personal technical-tactical ability (0.0369)
		a2-2 Opponent’s athlete’s personal technical-tactical characteristics (0.0327)
		a2-3 Opponent’s athlete’s physical condition (0.0227)
		a2-6 Opponent’s athlete’s physical fitness (0.0112)
		a2-7 Opponent’s athlete’s recent competitive state (0.0281)
		a2-8 Opponent’s athlete’s self-regulation ability (0.0112)
		a2-10 Opponent’s athlete’s age structure (0.0035)
		a2-11 Opponent’s athlete’s competition experience (0.0035)
		a2-12 Opponent’s athlete’s injury status (0.0035)
	A3 Athlete team collaboration (0.1358)	a3-1 Relationship among own athletes (0.0165)
		a3-2 Teamwork among own athletes (0.0165)
		a3-3 Own team’s common tactical approach (0.0350)
		a3-4 Own team’s lineup combination performance (0.0281)
		a3-5 Opponent’s common tactical approach (0.0327)
		a3-6 Opponent’s key players’ teamwork (0.0165)
		a3-7 Opponent’s lineup combination performance (0.0165)
B Tactical decision-making system (0.3125)	B1 Own coach’s competence (0.1852)	b1-1 Coach’s professional knowledge reserve (0.0227)
		b1-2 Coach’s decision-making style (0.0227)
		b1-3 Coach’s tactical style (0.0281)
		b1-4 Coach’s personality and psychological traits (0.0073)
		b1-5 Coach’s understanding of rules and officiating (0.0112)
		b1-6 Coach’s understanding of match patterns (0.0227)
		b1-7 Coach’s understanding and trust in athletes (0.0227)
		b1-8 Coach’s specialized technical-tactical competence (0.0327)
		b1-9 Coach’s information gathering ability (0.0227)
		b1-10 Coach’s authority (0.0165)
		b1-11 Coach’s experience in leading teams (0.0350)
	B2 Coach team collaboration(0.1111)	b2-1 Coach’s team communication (0.0112)
		b2-2 Coach’s team collaboration (0.0073)
		b2-3 Coach’s team innovation ability (0.0165)
C Match evaluation system (0.0625)	C1 Referee officiating level (0.0370)	c1-1 Referee’s control over the match (0.0073)
		c1-3 Referee’s professional ethics (0.0035)
	C2 competition organization (0.0124)	c2-1 Rationality of match time (0.0035)
		c2-2 Rationality of match scheduling (0.0008)
D Organizational and information support system (0.1875)	D1 Team management (0.0617)	d1-1 Team spirit (0.0165)
		d1-2 Importance of the match (0.0073)
	D2 Information collection and analysis (0.0864)	d2-1 Information collection methods and approaches (0.0281)
		d2-2 Information collection and analysis methods (0.0165)
		d2-3 Information integration (0.0165)

The different colors in Table are used to distinguish the four primary indicator systems.

For clarity, the indicator system determined through the Delphi method is referred to as the “theoretical-level indicator system,” while the backtracked and refined system is referred to as the “practical-level indicator system.”

#### 3.3.2 Questionnaire survey validation

To verify the consistency between the expert-refined indicator system and grassroots coaches’ perceptions of pre-match tactical decision-making, this study employed a questionnaire survey method to collect data on the importance ratings of various influencing factors from grassroots coaches. The mean scores for each indicator in the grassroots sample were calculated, and the ranking of indicators based on these scores was formed. Expert data were obtained from the importance ratings in the practical-level indicator system for the same indicators, from which the expert ranking was also determined.

Next, Spearman’s rank correlation coefficient (*ρ*) was calculated to examine the consistency between the indicator rankings of the expert group and those of the grassroots coach group. Because the purpose of this validation step was to compare the relative priority of indicators rather than to estimate a latent measurement structure, Spearman’s rank correlation was appropriate for the ordinal ranking data generated from the importance ratings. The analysis was used to assess the ranking consistency and practical applicability of the expert-refined indicator system.

The Spearman correlation coefficient is a non-parametric statistical method, also known as the rank correlation coefficient or ordinal correlation coefficient. Its calculation equation is:


$$\rho =1-\frac{6\sum d_{i}^{2}}{n(n^{2}-1)}$$

Where di represents the difference between the ranks of the same indicator in the two groups, and n represents the number of indicators included in the ranking. The Spearman rank correlation coefficient (*ρ*) ranges from [−1,1] and is used to measure the monotonic relationship between two variables (Spearman, 2010). Unlike the Pearson correlation coefficient, Spearman’s *ρ* is based on the ranks (order) of the data rather than the raw values, making it more robust to outliers and non-normally distributed data, and especially suitable for analyzing ordinal categorical variables or nonlinear relationships (Spearman, 1904). In this study, the correlation analysis for first-level, second-level, and third-level indicators was based on the number of indicators in each corresponding level as *n*.

The results showed that the *ρ-*values for the first-level indicators ranged from [0.653, 0.872] (Table 9). The lowest consistency level was for “C Competition Evaluation System,” which had a moderate consistency level; the highest was for “A Tactical Decision-Making Execution System,” which had an extremely strong consistency level. The average *ρ* for the first-level indicators was 0.755 (strong consistency), with all *p*-values < 0.01. For the second-level indicators, the *ρ* values ranged from [0.512, 0.892] (Table 10), with an average *ρ* of 0.724 (strong consistency level), and all p-values < 0.05. Among them, the highest consistency was for “A1 Own Athletes’ Strength,” which had an extremely strong consistency level, while the lowest was for “C2 Competition Organization,” which had moderate consistency. This was due to the experts’ relatively low attention to competition organization, while grassroots coaches, due to limited athlete levels, place greater emphasis on the rationality of competition organization.

**Table 9:** Spearman correlation coefficients for primary indicators.

**Indicator**	**Spearman’s ρ**	**p-value**	**Consistency levels**
A Tactical execution system	0.872	<0.001***	Very Strong
B Tactical decision-making system	0.785	<0.001***	Strong
C Match evaluation system	0.653	0.002**	Moderate
D Organizational and information support system	0.712	<0.001***	Strong

*p < 0.05, **p < 0.01, ***p < 0.001.

**Table 10:** Spearman Correlation Coefficient Statistics for Secondary Indicators.

**Indicator**	**Spearman’s ρ**	**p-value**	**Consistency levels**
A1 Own athlete’s strength	0.892	<0.001***	Very strong
A2 Opponent athlete’s strength	0.763	<0.001***	Strong
A3 Athlete team collaboration	0.701	<0.001***	Strong
B1 Own coach’s competence	0.815	<0.001***	Very strong
B2 Coach team collaboration	0.687	0.001**	Moderate
C1 Referee officiating level	0.634	0.003**	Moderate
C2 Competition organization	0.512	0.018*	Moderate
D1 Team management	0.723	<0.001***	Strong
D2 Information collection and analysis	0.798	<0.001***	Strong

*p < 0.05, **p < 0.01, ***p < 0.001.

For the third-level indicators, the overall average *ρ* was 0.73 (*p* < 0.001), with a 95% confidence interval of [0.698, 0.762], indicating strong consistency. Among them, the *ρ* for “C2-2 (Competition Schedule Reasonability),” “A2-11 (Opponent Athletes’ Competition Experience),” and “B2-3 (Coach Team’s Innovation Capacity)” were 0.38, 0.42, and 0.45, respectively, indicating weak consistency. The reason for this is that coaches of different levels paid varying degrees of attention to these three indicators. Grassroots coaches gave higher ratings to “C2-2″ than experts, while experts rated “A2-11″ and “B2-3″ higher than grassroots coaches. The experts invited in this study were all head coaches of provincial-level volleyball teams with senior or higher qualifications. Compared to grassroots coaches, they paid more attention to the competition experience of the opposing athletes and the innovation capacity of the coaching team, while they gave less attention to the reasonableness of the competition schedule, as they had already arranged training that adapted to various competition schedules and difficulties.

Overall, the indicator system, after expert backtracking validation, demonstrated a high level of order consistency with the actual pre-match tactical decision-making factors of grassroots coaches, reflecting a strong alignment in practical perceptions between the backtracking experts and grassroots coaches.

At the level of core influencing factor indicators, this study statistically summarized the grassroots coaches’ questionnaire data, extracting the top ten entries based on the mean rankings of third-level indicators. These entries were then compared with the top ten entries based on the weight rankings from both the theoretical and practical levels (post-backtracking) to examine the overlap and differences in key items.

As shown in Table 11, the top ten items in the theoretical and practical levels were completely consistent. Among the top ten items in the grassroots coaches’ sample, seven items overlapped with the aforementioned items, resulting in a 70% overlap rate. The differences mainly manifested as follows: grassroots coaches placed relatively more emphasis on factors such as athletes’ physical fitness, the performance of their team lineup combination, and the athletes’ recent competitive status. In contrast, experts and high-level coaches focused more on athletes’ individual technical and tactical abilities, characteristics, and information on the opponent’s common tactical plays.

**Table 11:** Comparison of the top ten tertiary indicators.

**Theoretical Level**	**Practical Level**	**Grassroots Coaches**
Athlete’s personal technical-tactical ability	Athlete’s personal technical-tactical ability	Athlete’s personal technical-tactical ability
Athlete’s team awareness	Athlete’s team awareness	Athlete’s team awareness
Athlete’s personal technical-tactical characteristics	Athlete’s personal technical-tactical characteristics	Athlete’s personal technical-tactical characteristics
Opponent athlete’s personal technical-tactical ability	*Opponent athlete’s personal technical-tactical ability*	Athlete’s tactical execution ability
Athlete’s tactical execution ability	Athlete’s tactical execution ability	Own team’s common tactical approach
Own team’s common tactical approach	Own team’s common tactical approach	Coach’s experience in leading teams
Coach’s experience in leading teams	Coach’s experience in leading teams	Coach’s specialized technical-tactical competence
Opponent athlete’s personal technical-tactical characteristics	*Opponent athlete’s personal technical-tactical characteristics*	*Athlete’s physical fitness*
Opponent’s common tactical approach	*Opponent’s common tactical approach*	*Own team’s lineup combination performance*
Coach’s specialized technical-tactical competence	Coach’s specialized technical-tactical competence	*Athlete’s recent competitive state*

In Table, yellow shading indicates indicators that appear in the theoretical/practical top-ten lists but not in the grassroots coaches’ top-ten list, while green shading indicates indicators that appear in the grassroots coaches’ top-ten list but not in the theoretical/practical top-ten lists. Unshaded items indicate overlapping indicators.

Overall, this difference suggests that grassroots coaches are more inclined to focus on “fundamentals and stability” when determining execution priorities in pre-match tactical decision-making, while high-level coaches tend to approach the process from a perspective of “oppositional relationships and tactical competition,” emphasizing targeted deployment and countermeasures.

### 3.4 Indicator analysis and comprehensive discussion

#### 3.4.1 Indicator analysis

1 First-level indicators

As shown in Table 8, the weight rankings for the four first-level indicators are as follows: A Tactical Decision-Making Execution System (0.4375), B Tactical Decision-Making Subject System (0.3125), D Organization and Information Support System (0.1875), C Competition Evaluation System (0.0625). Among these, the Tactical Decision-Making Execution System ranks first, reflecting the central role of athletes as the key force in a match and the most important factor determining the outcome of the game. Pre-match decisions are designed to ensure the team competes successfully and maximizes overall team strength, which is a dynamic process of reasonably allocating the team’s objective resources. Athletes are the main executors of tactical decisions, and the coach’s tactical decisions can only be realized through effective execution by the athletes.

The Tactical Decision-Making Subject System ranks second, highlighting the significant influence of the coach’s own qualities and team collaboration on pre-match tactical decisions. Modern volleyball is a contest of comprehensive strength between teams, not only involving the athletes’ competitive abilities but also the coach’s decision-making and command abilities, professional knowledge, and communication and collaboration skills (Kaya, 2014; Harvey et al., 2015). While individual preferences may exist in the coach’s team when developing a game plan, collective rationality tends to emerge in group decision-making based on pre-set goals, ultimately leading to the formulation of a relatively satisfactory game plan.

The Organization and Information Support System and Competition Evaluation System have a relatively smaller impact on the actual decision-making process, but their importance should not be overlooked. Among these, referees play an indispensable role in competitive sports, with their ability to apply the rules being crucial for the fairness and smooth progression of the match (Cipriano et al., 2019). Information collection and analysis serve as the foundation for pre-match tactical decision-making. A commonly used method is match analysis, which is one of the most important ways to gather information about both the coach’s and the opponent’s team (Fernandez-Echeverria et al., 2017). With advancements in artificial intelligence, algorithms, and big data technologies, coaches and researchers have further enhanced their ability to collect data in the competitive environment (McGillick et al., 2024).

2 Second-level indicators

The top four second-level indicators are as follows: A1 Own Athletes’ Strength (0.2099), B1 Own Coach’s Qualities (0.1852), A2 Opponent Athletes’ Strength (0.1605), A3 Athlete Team Collaboration (0.1358). As shown, the core factors influencing a coach’s pre-match tactical decision-making are the strength of their own athletes, team collaboration, and the coach’s qualities. However, volleyball is a process full of constraints and countermeasures, and the strength of the opposing athletes also plays a significant role in influencing the coach’s pre-match tactical decisions. Research shows that the five key aspects of an athlete’s performance during the match—height, power, accuracy, overall skill, and mental strength—are the main factors for success in modern volleyball. The orderly combination and comprehensive effect of these factors have a deeper impact on the outcome of the match (Chen et al., 2004). As opponents, the strength of the opposing athletes, in the form of “static information,” influences the coach’s lineup and tactical arrangements.

Moreover, as the commander and decision-maker of the match, the coach’s tactical decisions directly impact the athletes’ individual abilities and the team’s overall strength. Studies indicate that the value of a tactical plan depends on whether the team can form a consistent understanding and execute it effectively. The coach’s leadership style, team cohesion, and coach-athlete relationship influence motivation and collaboration, which in turn affect the quality of tactical execution and team performance (Mageau and Vallerand, 2003; Lafrenière et al., 2011; Vella et al., 2013). The coach’s leadership behavior is significantly positively correlated with team effectiveness, while authoritarian behavior negatively impacts team effectiveness (Jawoosh et al., 2022). A coach’s authority is essential for the successful execution of the game plan (Fiander et al., 2023). As a key component of a high-level volleyball team’s core competitive ability, the coach plays a crucial role in achieving victory.

1. 3 Third-level indicators

The top four third-level indicators are as follows: a1-1 Athlete’s Individual Technical and Tactical Ability (0.0388), a1-9 Athlete’s Team Awareness (0.0381), a1-2 Athlete’s Individual Technical and Tactical Characteristics (0.0369), a2-1 Opponent Athlete’s Individual Technical and Tactical Ability (0.0369).

On a micro level, volleyball is a contest of individual technical and tactical abilities between opposing athletes, making this the core factor that determines the outcome of the match. On a meso level, volleyball is a competition between the athletes of two teams, which requires athletes to possess not only excellent individual technical and tactical abilities but also strong team collaboration skills. From the perspective of team reasoning theory (Colman et al., 2008), due to the constraints of the game rules and the interdependence between team members, a team’s and individuals’ benefits can only be maximized when collective action and individual action on the court are integrated.

On a micro level, volleyball is a contest of individual technical and tactical abilities between opposing athletes, which is the core factor determining the outcome of the match. On a meso level, volleyball is a competition between the athletes of two teams on the court. This requires the coach to scientifically arrange lineup combinations and tactical strategies based on their athletes’ individual technical and tactical characteristics and the opponent’s abilities.

Additionally, a1-12 Athlete Age Structure (0.0008) and c2-2 Competition Schedule Reasonability (0.0008) are the two indicators with the lowest weights, indicating that for high-level teams, the athlete’s age structure and the reasonableness of the competition schedule have less impact on the coach’s pre-match tactical decisions. This is because competitive athletes tend to retire after reaching a certain age due to physical decline and injuries (Ronkainen et al., 2013; Koch et al., 2021). Organizers of competitions generally consider the reasonableness of the match schedule, and high-level teams incorporate training that adapts to various match schedules and difficulties during their regular practice sessions.

#### 3.4.2 Comprehensive discussion

1 Analysis of differences between theoretical-level and practical-level indicator systems

The research, based on the two indicator systems established through the Delphi method and expert backtracking, reveals the differences and complementarities between the theoretical and practical levels in terms of their functional orientation. The indicator system developed using the Delphi method mainly follows four principles: scientificness, systematization, comprehensiveness, and hierarchy. It is based on the consensus achieved by multiple experts’ knowledge and experience, emphasizing completeness and systematization, and focusing on theoretical soundness. In contrast, the indicator system refined through the expert backtracking method is grounded in actual competition and personal experience. By analyzing and distilling core factors with high operability and quantifiable characteristics, it highlights a practical orientation and application value, emphasizing usability and practicality.

The differences between the two systems reflect the transition from a “cognitive framework” to an “action guide.” The theoretical-level indicator system mainly explains the global influencing factors on volleyball coaches’ pre-match tactical decision-making, such as how the competition environment—such as the natural environment and unsportsmanlike behavior from spectators—affects athletes’ performance, and in turn, influences the coach’s tactical strategy and lineup decisions. For instance, low oxygen levels and cold climates in high-altitude environments not only negatively affect athletes’ physiological functions and performance but may also prevent some athletes from maintaining optimal performance or completing match tasks. Generally, teams from high-altitude areas tend to perform better in such environments, giving them a higher chance of winning (McSharry, 2007). When competing in or against high-altitude teams, coaches must make targeted decisions in response to these specific environmental conditions. Additionally, unsportsmanlike behavior from spectators primarily impacts the game through noise interference and disruptions to the match flow. Over time, the support of spectators has been seen as a significant home-court advantage (Marcelino et al., 2012; Oldfield et al., 2026) and it can even influence referees’ decisions, often leading them to favor the home team (Unkelbach and Memmert, 2010). Such factors also impact a coach’s pre-match lineup decisions.

While these indicators theoretically influence volleyball coaches’ pre-match tactical decisions, in practice, the impact of certain indicators may be lower due to variations in coach levels, competition tiers, and match environments. The Delphi method emphasizes the systematization and completeness of theoretical explanations, whereas the expert backtracking method focuses on the efficiency and practicality of operational aspects. The two methods are not in opposition but together form a logical loop that bridges theory and practice. The Delphi method provides the theoretical foundation for selecting indicators in the backtracking process, helping to avoid the limitations of empiricism, while the backtracking method validates the applicability of theoretical assumptions through practice, promoting the dynamic optimization of the theoretical model.

2 Practical value and limitations of the indicator system

This study, based on the indicator system constructed through the Delphi method, aims to identify those “visible but unclear” influencing factors in volleyball coaches’ pre-match tactical decision-making, presenting their multidimensional and interconnected structural features. This provides a relatively complete variable framework for subsequent research and pre-match tactical design. Overall, it leans more toward a “consensus-driven” indicator system. Building on this, we further streamlined the third-level indicators through the expert backtracking process, forming a “practice-oriented” indicator system that emphasizes operability, making it easier for coaches to quickly focus on key factors and adopt a checklist-based thinking approach during pre-match preparation.

From an application perspective, the hierarchical indicator system developed in this study is not only a way to present influencing factors and their weights, but can also be translated into an operational framework for coaches’ pre-match preparation. Primary indicators help coaches identify the main areas of attention, such as tactical execution, decision-making actors, match evaluation, and organizational and information support. Secondary indicators further specify the information collection and analysis modules within each area. Tertiary indicators can serve as concrete checklist items for identifying athlete status, opponent characteristics, lineup combinations, information preparation, and the basis for tactical execution. Based on this structure, high-level teams can use the system to optimize the focus and sequence of their pre-match information collection, such as gathering more targeted intelligence on the opponent, assessing their own team’s status, and identifying key matchups, thereby reducing omissions and fragmentation during decision-making. Grassroots coaches can also use the system as a reference framework for training arrangements and match strategy selection, fostering more intentional development of athletes’ basic technical-tactical abilities, team coordination, and tactical execution habits during daily training.

However, there are several limitations to this study that should be noted:

1. All experts and coaches involved in the consultation and validation were male and were recruited from the Chinese volleyball coaching context. Therefore, the final indicator hierarchy may partly reflect the training organization, coaching decision-making habits, and competition preparation routines in Chinese volleyball. Although women’s volleyball coaching contexts were covered to some extent, no subgroup comparison was conducted between men’s and women’s team coaching contexts. The system was also not independently validated in youth development programs or across elite, collegiate, and grassroots competitive levels. Therefore, the structural stability, weight distribution, and practical applicability of the indicator system should be further examined across men’s and women’s team contexts, youth development settings, competitive levels, international coaching contexts, and different cultural backgrounds.
2. The indicator weights were mainly derived from the judgments of the current expert panel and may have been influenced by expert composition, coaching background, and sample characteristics. Although the Delphi consensus, coefficients of variation, Kendall’s W, and the complementary check in the PC matrix helped improve the consistency of the weighting procedure, no formal sensitivity analysis of the indicator weights was conducted in this study.
3. The practical-level indicator system was developed through expert backtracking and questionnaire-based consistency testing. The backtracking evaluation was mainly used as a pre-screening procedure for contextual applicability, and the I-CVI ≥ 0.80 threshold was treated as an exploratory criterion for practical relevance rather than as final validity evidence. In addition, the grassroots coach survey included 67 valid responses for a practical-level system containing 64 items. This sample size is limited relative to the number of indicators and may affect the stability of the average priority rankings and consistency results. Therefore, the questionnaire findings should be interpreted as preliminary evidence of practical applicability and should be further examined in larger and more diverse coaching samples.

Future research can proceed in three directions:

1. Expand the sample sources to include female coaches, international coaches and experts, youth development programs, and coaches working at elite, collegiate, and grassroots competitive levels. Future studies should conduct subgroup comparisons between men’s and women’s team coaching contexts and across different competitive levels. Cross-gender, cross-cultural, cross-age-group, and cross-level validation is needed to examine the stability of the indicator structure, weight distribution, and practical applicability. With larger samples, future research should further clarify whether the indicators are formative or reflective in nature and, where appropriate, apply CFA, structural equation modeling, factor analysis, or multi-group validation to examine structural stability and reduce potential conceptual redundancy.
2. Integrate longitudinal match performance data, process data, and performance analytics to examine the sensitivity of indicator weights, calibrate key items empirically, and assess whether the indicator system can predict match outcomes or coaching effectiveness over time. Future studies may also use machine learning methods to update indicator importance dynamically based on accumulated match and coaching data, thereby moving from expert-consensus weights toward data-calibrated and context-sensitive weights.
3. Develop and pilot-test more operational coaching tools based on the practical-level indicator system, such as pre-match information collection checklists, tactical decision-support templates, coach education frameworks, or digital assistance systems. Future work should examine the usability, feasibility, and decision-support value of these tools in real coaching settings. Before wider application, the expert backtracking procedure, the I-CVI screening threshold, and the questionnaire-based consistency findings should also be re-examined with larger and more diverse coaching panels.

## 4 Conclusion

This study, based on systems theory, sports competition theory, and sports training theory, utilized the Delphi method and Precedence Chart (PC) method to construct an indicator system for volleyball coaches’ pre-match tactical decision-making factors. The system includes 6 first-level indicators, 15 second-level indicators, and 72 third-level indicators, systematically revealing the complexity of volleyball coaches’ pre-match tactical decisions at the theoretical level.

To assess the alignment of the system with real-world pre-match tactical decision-making scenarios, further expert backtracking evaluations were conducted to filter and streamline the third-level indicators, resulting in a practical-level indicator system. This final system retains four first-level indicators, nine second-level indicators, and 51 third-level indicators, making the indicator framework more closely aligned with the key concerns in coaches’ actual decision-making processes.

To verify the consistency between the constructed indicator system and the actual influencing factors in pre-match tactical decision-making, expert backtracking was used to identify key indicators that have a genuine impact in practice. These include 4 first-level indicators, 9 second-level indicators, and 51 third-level indicators. On this basis, a questionnaire survey was conducted to test the real-world applicability of the practical-level indicator system among grassroots coaches. The results showed that the average Spearman correlation coefficients for the first, second, and third-level indicators all indicated strong consistency, suggesting stable monotonic relationships between the levels of indicators. This reflects a high level of agreement between experts and grassroots coaches in their practical understanding.

This indicator system not only provides a theoretical framework and data support for volleyball coaches in areas such as pre-match planning, intelligence analysis, and strategy formulation, but also serves as an important reference for coaching decisions in other team sports. This study, to some extent, broadens the scope of sports competition theory and offers new perspectives for academic discussions in related fields.

## Data Availability

The original contributions presented in the study are included in the article/Supplementary material, further inquiries can be directed to the corresponding author/s.

## References

[ref1] Aagaard H. Scavenius M. Jørgensen U. (1997). An epidemiological analysis of the injury pattern in indoor and in beach volleyball. Int. J. Sports Med. 18, 217–221. doi: 10.1055/s-2007-972623, 9187978

[ref2] Ashford M. Abraham A. Poolton J. (2021). Understanding a player’s decision-making process in team sports: a systematic review of empirical evidence. Sports 9:65. doi: 10.3390/sports9050065, 34067590 PMC8156213

[ref3] Aughey R. J. Hammond K. Varley M. C. Schmidt W. F. Bourdon P. C. Buchheit M. . (2013). Soccer activity profile of altitude versus sea-level natives during acclimatisation to 3600 m (ISA3600). Br. J. Sports Med. 47, i107–i113. doi: 10.1136/bjsports-2013-092776, 24282196 PMC3903145

[ref4] Avugos S. (2024). Home advantage and the influence of officiating decisions. Sci. J. Sport Perform. 3, 188–219. doi: 10.55860/dubo8273

[ref5] Bilalić M. Gula B. Vaci N. (2021). Home advantage mediated (HAM) by referee bias and team performance during covid. Sci. Rep. 11:21558. doi: 10.1038/s41598-021-00784-8, 34732742 PMC8566522

[ref6] Callow N. Smith M. J. Hardy L. Arthur C. A. Hardy J. (2009). Measurement of transformational leadership and its relationship with team cohesion and performance level. J. Appl. Sport Psychol. 21, 395–412. doi: 10.1080/10413200903204754

[ref7] Chen T. Chu B. Qian Y. (2004). Research on the main factors for victory in volleyball and their orderly combination. J. Beijing Sport Univ. 3, 432–433.

[ref8] Chen Y. Li W. Wang H. Yang H. (2024). Construction of physical activity promoting indicators system for older adults with subjective cognitive decline using Delphi method. BMC Public Health 24:3206. doi: 10.1186/s12889-024-20762-9, 39558309 PMC11575052

[ref9] Cipriano P. Martins P. Duarte R. Preto L. Peixoto C. Moreira M. (2019). Investigation on soccer referees: a narrative review. Pol. J. Sport Tour. 26, 20–28. doi: 10.2478/pjst-2019-0016

[ref10] Colman A. M. Pulford B. D. Rose J. (2008). Collective rationality in interactive decisions: evidence for team reasoning. Acta Psychol. 128, 387–397. doi: 10.1016/j.actpsy.2007.08.003, 17868630

[ref11] Coutinho D. Gonçalves B. Wong D. P. Travassos B. Coutts A. J. Sampaio J. (2018). Exploring the effects of mental and muscular fatigue in soccer players’ performance. Hum. Mov. Sci. 58, 287–296. doi: 10.1016/j.humov.2018.03.004, 29549745

[ref12] Davis L. L. (1992). Instrument review: getting the most from a panel of experts. Appl. Nurs. Res. 5, 194–197. doi: 10.1016/S0897-1897(05)80008-4

[ref13] de Leeuw A. W. van Baar R. Knobbe A. van der Zwaard S. (2022). Modeling match performance in elite volleyball players: importance of jump load and strength training characteristics. Sensors 22:7996. doi: 10.3390/s22207996, 36298347 PMC9610012

[ref14] Debien P. B. Mancini M. Coimbra D. R. de Freitas D. G. Miranda R. Bara Filho M. G. . (2018). Monitoring training load, recovery, and performance of Brazilian professional volleyball players during a season. Int. J. Sports Physiol. Perform. 13, 1182–1189. doi: 10.1123/ijspp.2017-0504 29584530

[ref15] Drikos S. Vagenas G. (2011). Multivariate assessment of selected performance indicators in relation to the type and result of a typical set in men’s elite volleyball. Int. J. Perform. Anal. Sport 11, 85–95. doi: 10.1080/24748668.2011.11868531

[ref16] Eklund P. Rusinowska A. De Swart H. (2007). Consensus reaching in committees. Eur. J. Oper. Res. 178, 185–193. doi: 10.1016/j.ejor.2005.11.012

[ref17] Feltz D. L. Chase M. A. Moritz S. E. Sullivan P. J. (1999). A conceptual model of coaching efficacy: preliminary investigation and instrument development. J. Educ. Psychol. 91, 765–776. doi: 10.1037/0022-0663.91.4.765

[ref18] Fernandez-Echeverria C. Mesquita I. González-Silva J. Claver F. Moreno M. P. (2017). Match analysis within the coaching process: a critical tool to improve coach efficacy. Int. J. Perform. Anal. Sport 17, 149–163. doi: 10.1080/24748668.2017.1304073, 37339054

[ref19] Fiander M. F. Stebbings J. Coulson M. C. Phelan S. (2023). The information coaches use to make team selection decisions: a scoping review and future recommendations. Sports Coach. Rev. 12, 187–208. doi: 10.1080/21640629.2021.1952812

[ref20] Fox J. L. Stanton R. Sargent C. Wintour S.-A. Scanlan A. T. (2018). The association between training load and performance in team sports: a systematic review. Sports Med. 48, 2743–2774. doi: 10.1007/s40279-018-0982-5, 30225537

[ref21] Gabbett T. J. (2016). The training-injury prevention paradox: should athletes be training smarter and harder? Br. J. Sports Med. 50, 273–280. doi: 10.1136/bjsports-2015-095788, 26758673 PMC4789704

[ref22] Gesbert V. Carrel J. Philippe R. A. Hauw D. (2016). Elite volleyball coaches’ experience using a statistical information system. Int. J. Perform. Anal. Sport 16, 612–632. doi: 10.1080/24748668.2016.11868913

[ref23] González-Silva J. Moreno-Domínguez A. Fernández-Echeverría C. Claver F. Moreno M. P. (2020). Characteristics of serve, reception and set that determine the setting efficacy in men’s volleyball. Front. Psychol. 11:222. doi: 10.3389/fpsyg.2020.00222, 32132957 PMC7040554

[ref24] Gründel A. Schorer J. Strauss B. Baker J. (2013). Does playing experience improve coaching? An exploratory study of perceptual-cognitive skill in soccer coaches. Front. Psychol. 4:129. doi: 10.3389/fpsyg.2013.00129, 23518523 PMC3604731

[ref25] Harvey S. Lyle J. W. B. Muir B. (2015). Naturalistic decision making in high performance team sport coaching. Int. Sport Coach. J. 2, 152–168. doi: 10.1123/iscj.2014-0118

[ref26] Hsu C. F. Sandford B. A. (2007). The Delphi technique: making sense of consensus. Pract. Assess. Res. Eval. 12, 1–8. doi: 10.7275/pdz9-th90

[ref27] Jawoosh H. N. Alshukri H. A. Kzar M. H. Kizar M. N. Ameer M. A. A. Razak M. R. A. (2022). Analysis of coaches’ leadership style and its impact on athletes’ satisfaction in university football teams. Int. J. Hum. Move. Sports Sci. 10, 1115–1125. doi: 10.13189/saj.2022.100602

[ref28] Jones R. L. Wallace M. (2005). Another bad day at the training ground: coping with ambiguity in the coaching context. Sport Educ. Soc. 10, 119–134. doi: 10.1080/1357332052000308792

[ref29] Jorm A. F. (2015). Using the Delphi expert consensus method in mental health research. Aust. N. Z. J. Psychiatry 49, 887–897. doi: 10.1177/0004867415600891, 26296368

[ref30] Kaya A. (2014). Decision making by coaches and athletes in sport. Procedia Soc. Behav. Sci. 152, 333–338. doi: 10.1016/j.sbspro.2014.09.205

[ref31] Koch M. Klügl M. Frankewycz B. Lang S. Worlicek M. Popp D. . (2021). Football-related injuries are the major reason for the career end of professional male football players. Knee Surg. Sports Traumatol. Arthrosc. 29, 3560–3568. doi: 10.1007/s00167-021-06684-8, 34370085 PMC8514381

[ref32] Lafrenière M.-A. K. Jowett S. Vallerand R. J. Donahue E. G. Lorimer R. (2011). Passion for coaching and the quality of the coach–athlete relationship: the mediating role of coaching behaviors. Psychol. Sport Exerc. 12, 144–152. doi: 10.1016/j.psychsport.2010.08.002

[ref33] Lynn M. R. (1986). Determination and quantification of content validity. Nurs. Res. 35, 382???386–382???386. doi: 10.1097/00006199-198611000-00017, 3640358

[ref34] Mageau G. A. Vallerand R. J. (2003). The coach–athlete relationship: a motivational model. J. Sports Sci. 21, 883–904. doi: 10.1080/0264041031000140374, 14626368

[ref35] Manyara A. M. Purvis A. Ciani O. Collins G. S. Taylor R. S. (2024). Sample size in multistakeholder Delphi surveys: at what minimum sample size do replicability of results stabilize? J. Clin. Epidemiol. 174:111485. doi: 10.1016/j.jclinepi.2024.111485, 39069013 PMC7617918

[ref36] Marcelino R. Afonso J. Moraes J. C. Mesquita I. (2012). Determinants of attack players in high-level men’s volleyball. J. Strength Cond. Res. 26, 3385–3391. doi: 10.1519/JSC.0b013e3182474269, 22207260

[ref37] Marcelino R. Sampaio J. Mesquita I. (2011). Effects of quality of opposition and match status on technical and tactical performances in elite volleyball. J. Sports Sci. 29, 733–741. doi: 10.1080/02640414.2011.552516, 21424980

[ref38] McGillick C. Towlson C. Barrett S. Toner J. (2024). Performance analysis in sport and soccer: past, present and future–narrative review. J. Sports Res. 11, 19–41. doi: 10.18488/90.v11i1.3915

[ref39] McSharry P. E. (2007). Effect of altitude on physiological performance: a statistical analysis using results of international football games. Br. Med. J. 335, 1278–1281. doi: 10.1136/bmj.39393.451516.AD, 18156225 PMC2151172

[ref40] Medeiros A. I. A. Mesquita I. Marcelino R. Palao J. M. (2014). Effects of technique, age and player’s role on serve and attack efficacy in high level beach volleyball players. Int. J. Perform. Anal. Sport 14, 680–691. doi: 10.1080/24748668.2014.11868751

[ref41] Myers N. D. Feltz D. L. Maier K. S. Wolfe E. W. Reckase M. D. (2017). Athletes’ evaluations of their head coach’s coaching competency. J. Sport Exerc. Psychol. 39, 261–276. doi: 10.1123/jsep.2017-0155, 16646358

[ref42] Myers N. D. Vargas-Tonsing T. M. Feltz D. L. (2005). Coaching efficacy in intercollegiate coaches: sources, coaching behavior, and team variables. Psychol. Sport Exerc. 6, 129–143. doi: 10.1016/j.psychsport.2003.10.007

[ref43] Okoli C. Pawlowski S. D. (2004). The Delphi method as a research tool: an example, design considerations and applications. Inf. Manag. 42, 15–29. doi: 10.1016/j.im.2003.11.002

[ref44] Oldfield J. Oldfield R. Holmes D. (2026). The effects of different types of crowd noise on penalty taking performance in football. Int. J. Sport Exerc. Psychol. 24, 67–84. doi: 10.1080/1612197X.2024.2432996

[ref45] Palao J. M. Hernández-Hernández E. (2014). Game statistical system and criteria used by Spanish volleyball coaches. Int. J. Perform. Anal. Sport 14, 564–573. doi: 10.1080/24748668.2014.11868743

[ref46] Paul C. L. (2008). A modified Delphi approach to a new card sorting methodology. J. Usability Stud. 4, 7–30. doi: 10.5555/2835577.2835579

[ref47] Peña J. Casals M. (2016). Game-related performance factors in four European men’s professional volleyball championships. J. Hum. Kinet. 53, 223–230. doi: 10.1515/hukin-2016-0025, 28149426 PMC5260591

[ref48] Polit D. F. Beck C. T. Owen S. V. (2007). Is the CVI an acceptable indicator of content validity? Appraisal and recommendations. Res. Nurs. Health 30, 459–467. doi: 10.1002/nur.20199, 17654487

[ref49] Ronkainen N. J. Ryba T. V. Nesti M. S. (2013). ‘The engine just started coughing!’—limits of physical performance, aging and career continuity in elite endurance sports. J. Aging Stud. 27, 387–397. doi: 10.1016/j.jaging.2013.09.001, 24300059

[ref50] Shang Z. (2023). Use of Delphi in health sciences research: a narrative review. Medicine 102:e32829. doi: 10.1097/MD.0000000000032829, 36800594 PMC9936053

[ref51] Spearman C. (1904). The proof and measurement of association between two things. Am. J. Psychol. 15, 72–101. doi: 10.2307/1412159 3322052

[ref52] Spearman C. (2010). The proof and measurement of association between two things. Int. J. Epidemiol. 39, 1137–1150. doi: 10.1093/ije/dyq191, 21051364

[ref53] Sun Z. Zhang F. (2025). Constructing an indicator system for factors influencing volleyball coaches' in-game tactical decision-making: a Delphi study. Front. Psychol. 16:1721316. doi: 10.3389/fpsyg.2025.1721316, 41602698 PMC12833297

[ref54] Surya M. Benson A. J. Balish S. M. Eys M. A. (2015). The influence of injury on group interaction processes. J. Appl. Sport Psychol. 27, 52–66. doi: 10.1080/10413200.2014.941512

[ref55] Teques P. Duarte D. Viana J. (2019). Coaches’ emotional intelligence and reactive behaviors in soccer matches: mediating effects of coach efficacy beliefs. Front. Psychol. 10:1629. doi: 10.3389/fpsyg.2019.01629, 31379667 PMC6647934

[ref56] Unkelbach C. Memmert D. (2010). Crowd noise as a cue in referee decisions contributes to the home advantage. J. Sport Exerc. Psychol. 32, 483–498. doi: 10.1123/jsep.32.4.483, 20733209

[ref57] Vella S. A. Oades L. G. Crowe T. P. (2013). The relationship between coach leadership, the coach–athlete relationship, team success, and the positive developmental experiences of adolescent soccer players. Phys. Educ. Sport Pedagog. 18, 549–561. doi: 10.1080/17408989.2012.726976

[ref58] Verhagen E. A. L. M. Van der Beek A. J. Bouter L. M. Bahr R. M. Van Mechelen W. (2004). A one season prospective cohort study of volleyball injuries. Br. J. Sports Med. 38, 477–481. doi: 10.1136/bjsm.2003.005785, 15273190 PMC1724865

[ref59] Wang X. Chen T. Zhang Y. Yang H. H. (2021). Implications of the Delphi method in the evaluation of sustainability open education resource repositories. Educ. Inf. Technol. 26, 3825–3844. doi: 10.1007/s10639-021-10452-z

[ref60] Wang Y. Tian D. (2016). A weighted assembly precedence graph for assembly sequence planning. Int. J. Adv. Manuf. Technol. 83, 99–115. doi: 10.1007/s00170-015-7565-5

[ref61] Xie L. Feng M. Cheng J. Huang S. (2023). Developing a core competency training curriculum system for emergency trauma nurses in China: a modified Delphi method study. BMJ Open 13:e066540. doi: 10.1136/bmjopen-2022-066540, 37130690 PMC10163488

[ref62] Ye B. Zhu H. Yang Z. He Z. Liu G. Pan H. . (2024). Construction and analysis of the physical fitness evaluation index system for elite male singles badminton players: based on Delphi and AHP methods. Life 14:944. doi: 10.3390/life14080944, 39202686 PMC11355487

[ref63] Zajonz D. Kittel A. Gropper H. Raab M. (2024). Emotional intelligence training for sports coaches: evaluation of a training approach to increase coaches’ emotional intelligence and reduce reactive behaviors. J. Appl. Sport Psychol. 36, 722–739. doi: 10.1080/10413200.2023.2296911

[ref64] Zamanzadeh V. Ghahramanian A. Rassouli M. Abbaszadeh A. Alavi-Majd H. Nikanfar A. R. (2015). Design and implementation content validity study: development of an instrument for measuring patient-centered communication. J. Caring Sci. 4, 165–178. doi: 10.15171/jcs.2015.017, 26161370 PMC4484991

